# Biochemical and histopathological changes in livers of rats poisoned with aluminum phosphide and treated with carrot extract

**DOI:** 10.5620/eaht.2023014

**Published:** 2023-06-30

**Authors:** Ali Husein Ali Al-Safar, Rafat A. Mohammed Jawad, Hussein Ali Khayoon, Mohenned A. Alsaadawi, Khalil Hassan Zenad, Zeayd Fadhil Saeed, Mansoor J. Alkhaled

**Affiliations:** 1 College of Veterinary Medicine, University of Al-Muthanna, Iraq; 2 College of Pharmacy, University of Al-Muthanna, Iraq; 3 College of Nursing, Al-Muthanna University, Iraq; 4 College of Veterinary Medicine, University of Baghdad, Iraq; 5 Nursing department, Technical Institute of Samawah, Al Furat Al Awsat Technical University, Iraq; 6 College of Veterinary Medicine, University of Al-Qadisiya, Iraq

**Keywords:** Aluminum Phosphide, Carrot Extract, biochemical, histopathological changes, liver enzymes

## Abstract

The experimental studies of Aluminum Phosphide (AP) poisoning in rats revealed several clinical and pathological signs such as hemorrhage, sinusoidal dilatation, bile stasis, centrilobular necrosis, Kupffer cell hyperplasia, infiltration by mononuclear cells, and fatty infiltration in the liver tissues. This paper aimed to show the impact of carrots on the toxic effect of AP on the livers of adult rats (female). To investigate some biochemical and histopathological changes effects of AP in rats, sixty white female rats were equally divided into four groups, the first group (G1) was administered orally with 3mg/kg/ body weight of AP, the second group (G2) was orally treated with AP and 10% carrot extract at the same time. The third group (G3) administrated 10% carrot extract only. The fourth (G4) group was the negative control and was treated with distilled water only. The experiments continued for a month at the animal house of the Veterinary Medicine College of Baghdad University. The results revealed that high levels of liver enzymes and bilirubin were induced in G1 with decreasing total protein levels. The pathological examination revealed the presence of marked proliferation of Kupffer cells in G1 livers. However, the G2 group showed slight infiltration of lymphocytes in sinusoids. The pathological changes in the livers of G3 group showed slight cloudy swelling in hepatocytes compared with the normal texture of hepatocytes in G4. The data of this experiment showed that treatment with carrot extract significantly decreases the elevation in the level of liver function enzymes in animal poisoned with AP. In addition, treatment with carrot extract reduces the severe damage in the hepatic tissue that occurred in rats treated with AP only. In general, it could be concluded that treatment with carrot extract provides a remedial effect against the hepatotoxicity that is resulted from exposure to AP.

## Introduction

Organic and inorganic poisoning can cause common pathological and biochemical disorders such as Potassium Nitrate toxicity, Lead poisoning [1], or chlorpyrifos and deltamethrin [2]. AP is a relatively inexpensive and widely used rodenticide. Also, AP can be widely used as a solid fumigant in preserving grains. There are many trade names of this substance such as Quickphos, Phostek, Alphos, Celphos, and Synfume. The reason behind the high percentage of poisoning in developing countries could be due to the absence of restrictions on this chemical material [3,4].

The poisoning effect relates to phosphine gas (PH3) which is considered a highly poisoning agent that can be formed when AP contact with either moisture or water or hydrochloric acid (HCL) inside the stomach [3–5]. Toxicity to humans is most typically associated with AP intake. However, toxicity by inhalation and absorption through the skin are also possibilities [5, 6]. As a result, systemic poisoning can be caused by diffusingPH3 through the digestive system and thus reaching different parts of the body [5]. Activation of PH3 could lead to inhibit cytochrome C oxidase, thus inhibiting oxidative phosphorylation and cell respiration. In addition, the reactive oxygen species (ROS) can be accumulated which is responsible for further cellular damage ultimately resulting in cellular death [6]. Therefore, the high oxygen-demanding organs such as the heart, lungs, kidneys, liver, and brain, are more susceptible to poisoning with phosphine which finally may lead to oxygen free radicals’ formation [7]. Acute AP poisoning is characterized by the presence of nausea, vomiting, abdominal discomfort, and hemodynamic instability which are all common symptoms [8,9]. Also, acute respiratory distress syndrome, hepatic, heart, and renal failure are all possible complications of AP poisoning [5,10,11].

Due to the lack of an effective antidote for AP poisoning, supportive treatment is the primary approach [12]. This necessitated trial of many drugs to obtain an antidote drug. For a long time, Magnesium sulfate (MgSO4), was used to treat AP poisoning as it has an antioxidant and cell membrane stabilizer effect. However, many researchers recorded opposite results [13]. Many studies have examined the changes in liver enzymes as an indicator of liver damage during chemicals or extract consumption [13]. A supportive theory of using MgSO4 relies on hypomagnesemia that resulted from AP poisoning [14,15]. However, other studies found that supplementing MgSO4 did not show any improvement from AP poisoning, and the MgSO4 levels were not affected [16]. Additionally, there are different antidotes to treat AP poisoning such as melatonin, boric acid, vitamin C and E supplements, coconut oil, and acetyl-L carnitine [3,17,18]. AP is widely used to prevent eating grains by pests and rodents through fumigation (19). The lethal dose of AP for adult individuals is between 0.15 and 0.5 grams of the substance [10,11].

A study by Eric et al., 2021 was done to evaluate some of the in vivo pharmacological actions of garlic extract against AP toxicity in rats. The data of this experiment indicated that animals exposed to AP toxicity showed congestion in main veins, organ hemorrhage, dilatation of sinusoids, mononuclear cell infiltration, and fatty infiltration in liver tissues on microscopic examination. In addition, AP when mixed with garlic extract at a variety of concentrations (250 mg/l and 500 mg/l), has been demonstrated to have a more toxic effect on the liver tissue and did not provide a remedial effect against AP poisoning [20]. Recently, herbal plants are widely used in different medicinal applications due to their clear effect in reducing the drawbacks of many infectious and non-contagious diseases [21]. β-Carotene is composed of two retinyl groups, and is broken down in the mucosa of the human small intestine by β-carotene 15,15'-monooxygenase to retinal, a form of vitamin A. β-Carotene can be stored in the liver and body fat and converted to retinal as needed, thus making it a form of vitamin A for humans and some other mammals. The carotenes α-carotene and γ-carotene, due to their single retinyl group (β-ionone ring), also have some vitamin A activity (though less than β-carotene), as does the xanthophyll carotenoid β-cryptoxanthin. All other carotenoids, including lycopene, have no beta-ring and thus no vitamin A activity (although they may have antioxidant activity and thus biological activity in other ways) [22].

This study aimed to investigate the effectiveness of carrot extraction in improving/or treating biochemical and pathological changes in the livers induced by AP poisoning of adult female rats.

## Materials and Methods

Animals and treatments: sixty adult female rats (Wistar albino aged eight weeks), weighing 250 ± 10 gm, were collected from the Animal House at the Veterinary Medicine College of Baghdad University for a month. The laboratory animals were stored in temperature-monitored rooms (24 ± 3 °C) and humidity (20-25%), and a 12h/12h light/dark period prior to being used in experimental procedures. To accommodate the workshop conditions, rats were allowed to participate in the experiment for one week. Rats were grouped into four groups each group consisting of 15 rats. The G1 group received only AP, the G2 group was administered with AP and 10% carrot extract, the G3 group received carrot extract (10%) [23] only while the last group, the G4, was the control group rats and were often given orally distilled water only. The used dose of AP in this study was 3 mg/kg body weight/daily dissolved in sterile water and given orally (b.w. is an abbreviation of body weight). The single lethal dose of AP that was previously recorded was 10 mg/kg body weight [24]. The administration continued for 90 days through the stomach drain. Carrot plant extracts were soaked in such a way that 10 grams of each ash vegetarian were consumed while drinking 100 ml of distilled water for two hrs. The extract was concentrated by filtering the mixture through Whatman filter paper, then centrifuged at 3000 rpm for ten minutes, then the filtrate was stored in a sterile bottle in the refrigerator at 4°C for future use [25]. For hematological examination, the animals' blood was collected after 90 days.

### Histopathological study

The liver was taken and fixed in 10% formalin, for staining with hematoxylin (basophilic nucleus stain) and eosin (eosinophilic cytoplasmic stain) (H & E) [26], to be examined under the light microscope (Leica, Germania) (26,27).

### Data analysis

The statistical program used in this study was GraphPad Prism 9 and the statistical analysis was One Ways ANOVA. The findings were described as mean ± S.E.M (standard error mean). A P-value of less than 0.05 was significantly important and is described in brackets.

## Results and Discussion

In the current study, rats were divided into four separate groups. G1 group received only AP (3 mg/ kg b.w./daily), G2 group was administered with AP and 10% carrot extract, G3 group received carrot extract only while the last group, G4, was the control group rats and were often given oral ingestion of distilled water only. The hematological examination of treated rats includes revealing the liver enzymes, bilirubin, and total protein concentrations in order to show the impact of AP administration and the counteract of carrot extract on AP poisoning.

### Biochemical aspect

**Figure 1.**
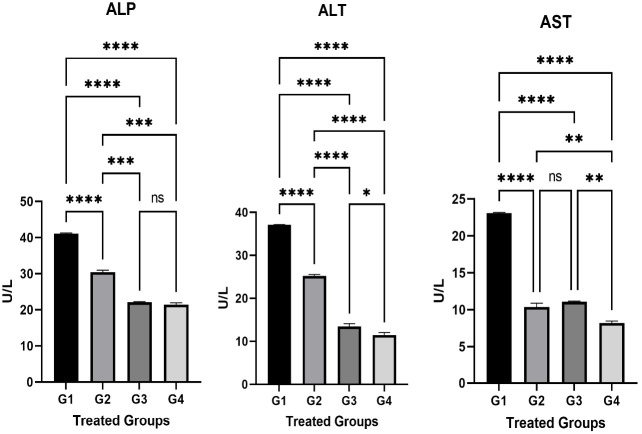
The relation of the liver enzymes in different treated groups. The liver enzymes were checked at the end of the experiment. These enzymes include ALP, ALT, and AST. The differences between the parameter concentrations were significant in all the treated groups. The statistical analysis was done using GraphPad Prism 9 program and the test was One Ways ANOVA and Dunnett's multiple comparisons test, P≤0.05.

A significant increase (p < 0.05) was recorded in the Alkaline phosphatase (ALP mg / dL) in G1 (Figure 1) received AP only and G2 treated with both AP and carrot extract compared to the control group G4. While there was no significant increase in G3 group that did not show any significant differences in comparison with G4 (Figure 1). Regarding Alanine Aminotransferase Tests (ALT), the tests showed a significant increase (p < 0.05) in G1, G2, and G3 compared to the control group (Figure 1). The tests of serum Aspartate Aminotransferase (AST) revealed that there is an increase in G1, G2, and G3 (Figure 1). The differences between G1 and G2 were statistically different (P < 0.05) in the above three tests. Therefore, it could be concluded that adding carrot extract to the AP poisoning group (G2) showed a significant decrease in tested liver enzyme levels compared to G1.

**Figure 2.**
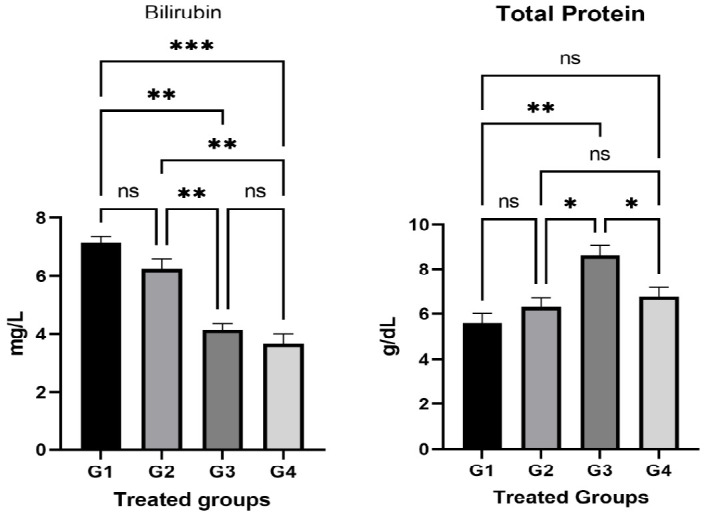
The relation of the liver Bilirubin and total liver protein in different treated groups. The results of liver functions (including Bilirubin and total liver protein) were statistically compared between groups by GraphPad Prism 9 program using One Way ANOVA and Dunnett's multiple comparisons test, P≤0.05.

The bilirubin test values increased significantly (P < 0.05) in G1 and G2 in relation to the control group (G4) (Figure 2). There was also an insignificant increase in G3. There was a significant increase (p < 0.05) in the total protein proportion of G3 compared to the control group (G4). However, there is an insignificant decrease in G1 and G2 compared to G4 (Figure 2).

### Histopathological aspect

**Figure 3.**
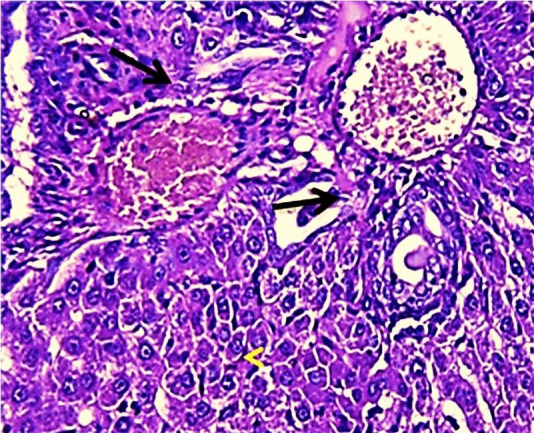
Histopathological section of liver of G1 rats. This figure showed marked proliferation of Kupffer cells and Expansion of blood sinusoid vacuolated hepatocytes (red arrow) (H&E) (400X).

**Figure 4.**
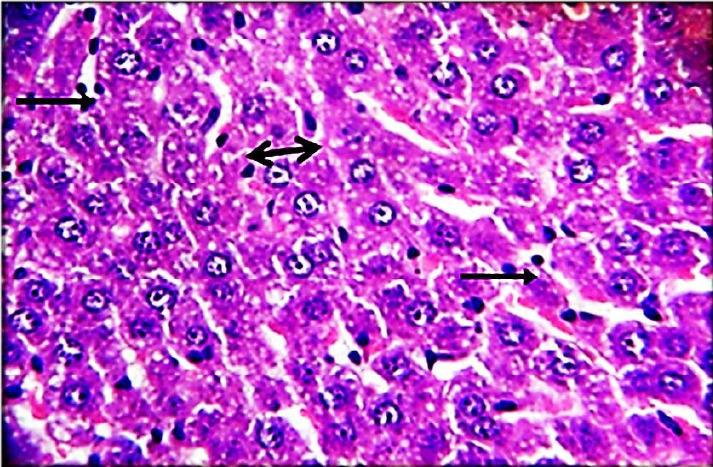
Histopathological section of liver of G2 rats. This figure showed marked proliferation of Kupffer cells (black arrow) with pyknotic (H&E)(400X).

**Figure 5.**
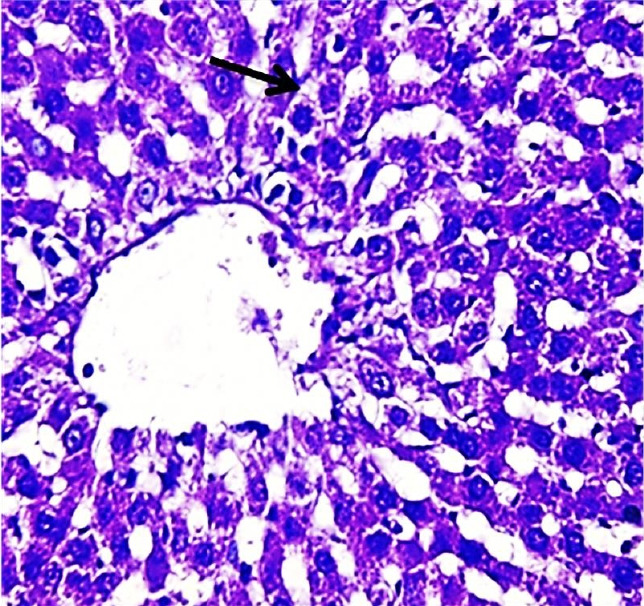
Histopathological section of liver of G3 rats. Livers showed slight cloudy swelling in hepatocytes (arrow) (H&E) (400X).

**Figure 6.**
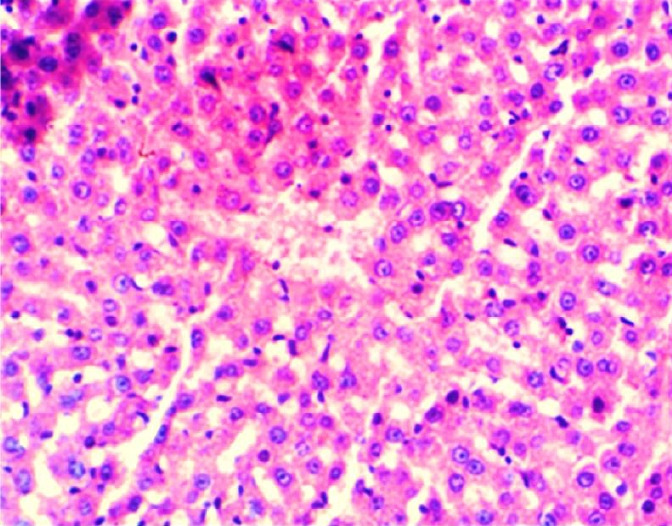
Histopathological section of liver of G4 rats. Livers showed normal texture in hepatocytes, and this is treated with distilled water (normal) (H&E) (200X).

The results of this study revealed the presence of different histopathological changes in the liver. These changes appeared in rats after ninety days post-treated by (AP) which showed marked proliferation of Kupffer cells with pyknotic and necrosis in hepatocytes. In addition, there is an aggregation of mononuclear cells (Figure 3). A significant proliferation of Kupffer cells with a minor infiltration of lymphocytes in the sinusoids was identified in the histopathological examination of the liver in group two (G2) (Figure 4). The changes in G3 were confined to slight cloudy swelling in hepatocytes (Figure 5&6).

Our study found that there was a significant increase in liver enzymes and bilirubin levels P≤0.05in G1 (AP poisoning group). However, an increase in the examined parameters in G2 was less than G1 treated with carrot extract and suffered from AP poisoning (Figure 1&2). This can be attributed to the escape of liver enzymes and bilirubin from hepatocytes to the intestine and bone in the G1 and G2 groups. Thus, this may refer to the impairment of liver function in these two groups due to AP poisoning. However, this effect is less in G2 than G1 which could obviously show how the carrot can minimize the AP poisoning to some extent.

The results showed a non-significant decrease in the total protein of G1 and G2 compared to G4. The dropping in total protein might be due to toxic liver injury, resulting in disturbances in the transport functions of the hepatocytes [26]. The total protein in G3 was significantly higher than in the control group. This can relate to the role of carrots in increasing the total protein production of hepatocytes.

In general, and due to the protective effect of carrot extracts on the liver, G2 showed a significant decrease (P≤0.05) in liver enzymes and bilirubin compared to G1.

The Histopathological study showed severe damage in the liver of G1 because of the free radical production induced by AP which is directly cytotoxic to hepatic tissue. The tissues can be damaged during prolonged oxidative stress. These findings are consistent with [27], who discovered that AP increased the number of apoptotic cells. AP can accumulate in the liver and cause oxidative damage to hepatic cell membranes, causing transaminase to release into the bloodstream [28]. While the carrot extract-treated group demonstrates only minor histopathological changes in the liver which could relate to the major organosulfur constituents of carrot that can inhibit the induction of free radicals and clearing of hydrogen peroxide [29]. In addition, carrot extract contains certain compounds that can induce the neutralization of oxygen used by living cells. These compounds are elements found in plants such as germanium and selenium [30].

## Conclusions

AP poisoning can lead to a significant increase in liver enzymes and bilirubin levels P≤0.05. AP poisoning rats treated with carrot extract can decline the rise in liver enzymes and bilirubin induced by AP. However, total protein levels dropped down in the AP-treated group which may relate to toxic liver injury, resulting in disturbances in the transport functions of the hepatocytes induced by AP. Adding carrot extract to the AP poisoning group (G2) showed a significant decrease in tested liver enzyme levels compared to G1. This clearly explains the positive impact of carrot extract on the treated groups. The Histopathological study showed severe damage in the liver of the AP poisoning group because of the free radical production induced by AP which is directly cytotoxic to hepatic tissue. While the carrot extract-treated group suffered from AP poisoning demonstrates only minor histopathological changes in the liver which could relate to the major organosulfur constituents of carrot that had a scavenging effect on hydrogen peroxide, and inhibited role for the chain of oxidation induced by hydrophilic radical initiation. In general, carrot extract can decrease the damage of liver cells from oxidative effects induced by AP, and that relates to its antioxidant effects.
